# Drivers of zoonotic spillover at human–domestic animal–wildlife interfaces: a multi-species model incorporating wildlife chronic stress

**DOI:** 10.1098/rsos.260184

**Published:** 2026-11-04

**Authors:** Erin Stafford, Junwen Guo, Peter Fransson, Åke Brännström, Alex D. Greenwood, Kyrre Kausrud, Henrik Sjödin

**Affiliations:** ^1^ Department of Epidemiology and Global Health, Umeå University, Umeå, Västerbotten County, Sweden; ^2^ Mathematics and Mathematical Statistics, Umeå University, Umeå, Västerbotten County, Sweden; ^3^ Zeeman Institute for Systems Biology & Infectious Disease Epidemiology Research, Mathematics Institute and School of Life Sciences, University of Warwick, Coventry, UK; ^4^ Interdisciplinary Center for Scientific Computing, Heidelberg University, Heidelberg, Baden-Württemberg, Germany; ^5^ Complexity Science and Evolution Unit, Okinawa Institute of Science and Technology Graduate University, Onna, Japan; ^6^ Advancing Systems Analysis Program, International Institute for Applied Systems Analysis, Laxenburg, Austria; ^7^ Department of Wildlife Diseases, Leibniz Institute for Zoo and Wildlife Research (IZW) in the Forschungsverbund Berlin e.V., Berlin, Germany; ^8^ Department of Veterinary Medicine, Freie Universität Berlin, Berlin, Germany; ^9^ Norwegian Veterinary Institute, Ås, Norway; ^10^ Department of Wildlife, Fish and Environmental Studies, Swedish University of Agricultural Sciences - Umea Campus, Umeå, Västerbotten County, Sweden

**Keywords:** mathematical modelling, sensitivity analysis, zoonotic spillover, wildlife stress

## Abstract

Zoonotic disease emergence at the wildlife–domestic animal–human interface results from a complex interplay of ecological, physiological and anthropogenic factors. Here, we have developed a multi-environment, multi-host compartmental model to identify key drivers of spillover risk across urban, rural and natural environments. The framework incorporates heterogeneous host populations, inter-species contacts and host movement, and includes wildlife chronic stress as one factor influencing susceptibility, transmissibility and recovery. Sensitivity analyses show that domestic animal populations at agricultural interfaces strongly amplify spillover risk through their recovery dynamics and per-contact transmission probabilities, while within-wildlife transmission across environments also shapes system dynamics. Chronic stress effects can further increase spillover risk, and the model indicates that reducing wildlife stress alongside improving domestic animal health and management can markedly lower the likelihood of cross-species transmission. These findings highlight the importance of maintaining ecological conditions that reduce chronic physiological stress in wildlife populations and integrating physiological and ecological data into epidemiological modelling. Such models can inform both bottom-up interventions targeting domestic animals and top-down strategies that sustain resilient wildlife communities and, thereby, mitigate zoonotic spillover.

## 1. Introduction

Many human health relevant infectious diseases have emerged through zoonotic spillover from epizootic transmission among wildlife species [1–3]. This pattern is not surprising given that viruses and other infectious agents have relied on biological hosts for billions of years. The occurrence of epizootic transmission among wildlife is therefore expected and not inherently concerning. Nonetheless, although spillover into humans is extremely rare, such events can occasionally lead to severe outcomes. This has gained relevance under increasing anthropogenic pressures on ecosystems, which may elevate epidemiological risks, underscoring the need to better understand the complex processes involved. For example, in the last half century, HIV/AIDS emerged from non-human primates in Africa and has led to more than 25 million deaths worldwide since being recognized in 1981 and remains an ongoing pandemic [4]. Emerging in the beginning of the twenty-first century from, probably, bats [5], epidemics of SARS and MERS have had mortality rates as high as 10–50% (depending on age) and approximately 50%, respectively, [6]. The COVID-19 pandemic has further underscored the risks of emerging pathogens, with SARS-CoV-2 demonstrating the ability to cross species barriers, including documented spillover into farmed mink populations and subsequent spillback into humans that generated new viral variants [7]. Considering the vast diversity and abundance of life on Earth, zoonotic outbreaks are exceptionally rare events. Wildlife should therefore not be viewed as a source of danger, but as an integral component of a healthy biosphere. However, anthropogenic stressors increasingly disrupt ecological systems, intensifying epizootic processes at a time of rising human population density, expanding domestic animal biomass and accelerating environmental change. Advancing our understanding of these processes is essential for anticipating and mitigating future zoonotic emergence and pandemic risk.

Avian influenza is a useful example that illustrates how interactions between wildlife, livestock and ecosystem disturbance can amplify these processes. Recently, highly pathogenic avian influenza (HPAI H5Nx), emerging in farmed birds and cycling back and forth between domestic and wild populations, is increasingly being viewed as a potential threat to human health [8–10]. Since January 2020, there have been 103 reported human infections and 11 deaths worldwide [8], mostly resulting from contact with infected birds [11]. Recurrent outbreaks, facilitated in part by the migratory patterns of wild birds, have been devastating both wildlife populations and livestock [11,12]. Recent reports also indicate avian influenza virus transmission from dairy cattle to humans, manifesting primarily as conjunctivitis rather than respiratory illness [13]. In the agricultural industry, mass deaths owing to disease or culling have resulted in substantial economic losses. For example, the 2022 outbreak of avian influenza led to the loss of 40 million animals and between 2.5 and 3 billion USD in economic losses in the United States alone [14]. The disease transmission pathway across a wildlife–domestic animal–human interface is not unique to avian influenza. Nipah virus encephalitis, Hendra virus disease, Menangle virus disease, Campylobacteriosis and Japanese encephalitis are all examples of zoonotic diseases with domestic intermediate hosts that facilitate the spread of zoonotic disease from wild reservoir hosts to humans [15].

The opportunities for spillover events along this pathway, and others, are also increasing owing to increased global trade and extensive land-use or land-cover changes, which include agriculturalization [16–22]. Moreover, environmental changes can lead to chronic stress in wildlife populations, which further amplify the risk of zoonotic spillover through the physiological and behavioural changes in wildlife that result [23,24]. Such changes not only reshape contact patterns but also directly affect animal populations, creating physiological conditions that further elevate spillover risk.

Chronic stress is a prolonged activation of the hypothalamic–pituitary–adrenal axis that can result from repeated or persistent environmental stressors [25,26], leading to the accumulation of allostatic load that may persist indefinitely. This sustained physiological state induces immunological and metabolic changes that increase host susceptibility, viral loads and shedding intensity and infection duration [24,27,28]. In addition to these internal effects, chronic stress can alter the behaviour of wildlife by driving animals to congregate in resource-rich areas or forcing them into closer proximity with domestic animals and humans [21,28].

Evidence of the harmful effects of chronic stress in wildlife populations on zoonotic spillover emphasizes the need for studying stress effects. Mathematical models are ideal for this as they allow for the exploration of dynamics which can be hard to track in empirical studies and for the prediction of the potential effects of, for instance, continued climate change and land-use changes. For example, Owen *et al.* [27] combined experimental studies with individual-based modelling to determine how food stress in American robin populations affected the risk of West Nile virus transmission to humans. This study showed that, even when the proportion of stressed birds was small, the increased viral loads and infection periods from host stress increased infection rates [27]. Other modelling studies have found that environmental stressors, such as increased temperatures [29] or mercury exposure [30], increase the spread of disease, while predation fear in populations may decrease disease transmission [31].

To place these studies in a broader context, we systematically assessed the extent to which existing models examine the role of chronic stress in zoonotic spillover specifically. We conducted a structured search in the Web of Science database through October 2024 using combinations of terms related to mathematical modelling, disease spillover and animal stress (full search strings are available in table S1 in the supplementary material). This search yielded a total of 1547 records. All articles were screened to assess their relevance and to determine whether they described a conceptual or quantitative model addressing the relationship between stress and disease spillover. The screening process involved reviewing titles, abstracts and, where necessary, full texts to ensure that potentially relevant studies were not overlooked. We identified only one study that explicitly modelled the effect of stress on infection dynamics: the agent-based model of West Nile virus that demonstrated how food stress in a reservoir host can elevate viraemia, extend infectious periods and increase mosquito infection rates [27]. While this represents an important advance, it is limited to a single vector–host system. To the best of our knowledge, similar studies looking at the effects of chronic stress in wildlife on the spread of disease between wildlife, domestic animals and humans have not been performed.

Here, we present a mathematical model that addresses the gap in understanding the role of chronic stress in zoonotic disease spillover along the wildlife-domestic-human interface. Previous studies have used compartmental models to investigate pathogen transmission within three-host wildlife–domestic animal–human systems [15]. Similarly, we consider a generalized three-host spillover framework, but focus on a different epidemiological setting by developing a spatial susceptible–infected–susceptible (SIS) model that incorporates chronic physiological stress as one mechanism through which environmental change can influence wildlife susceptibility, transmission and recovery. To reflect real-world complexity, we extend the model into a patch network encompassing urban, rural and natural environments, allowing for population movement and heterogeneous contact patterns across landscapes. Through detailed sensitivity analyses, we quantify how chronic stress and changing inter-species interactions can affect the risk of zoonotic disease transmission. Our findings reveal key drivers of zoonotic spillover and highlight the environmental and biological variables most critical for estimating location-specific spillover risk.

## 2. Methods

To study key drivers, including stress on spillover risk, we developed a generalizable mathematical model of pathogen transmission among humans, domestic animals and wildlife across urban, rural and natural environments. The model incorporates wildlife stress as a modifier of susceptibility, transmissibility and recovery, extending beyond simple host–pathogen systems to capture cross-species interactions in complex landscapes (figure 1). We then applied local and global sensitivity analyses to the model in order to quantify how wildlife stress and other factors influence zoonotic spillover risk and to identify key transmission drivers.

**Figure 1.**
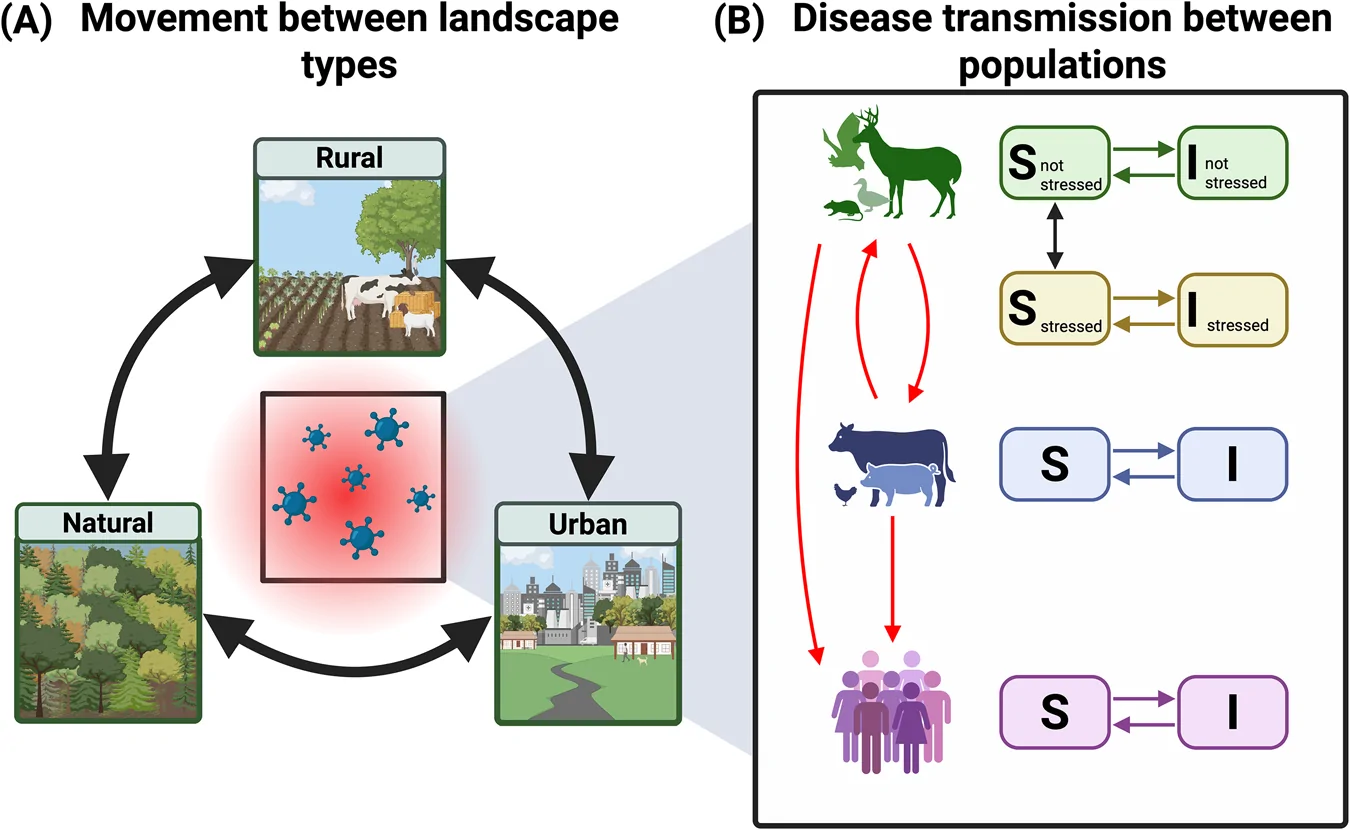
Flow diagram of the stress-induced spillover model. (A) Host movement among urban, rural and natural environments. (B) Compartmental disease transmission structure within each environment, showing susceptible (S) and infected (I) states for humans, domestic animals and stressed and non-stressed wildlife. Arrows between S and I states indicate transitions between disease states over time, while lines between host populations denote transmission routes (Created in BioRender. Stafford, E. (https://BioRender.com/03esivw) is licensed under CC BY 4.0.).

### 2.1. Mathematical model overview

We modelled three categories of hosts—humans (H), domestic animals (D), including livestock and pets, and wildlife (W)—and the transmission of a hypothetical pathogen among these host populations. We represent transmission dynamics through a compartmental SIS framework, which provides a general representation of directly transmitted zoonotic infections in which reinfection may occur following recovery [32–34] and has been applied to wildlife-associated diseases including bovine tuberculosis in African buffalo and rodent-borne Bartonella infections [35,36]. We adopt this generalized SIS framework to focus on the effects of chronic stress on transmission processes rather than disease-specific immune dynamics, allowing the model to remain broadly applicable across wildlife–domestic animal–human systems. Moreover, we omit birth and death in this model so as to increase the generality of our framework because including demographic processes would introduce additional, widely varying system-specific parameters. The landscape consists of three interconnected environments, urban (U), rural (R) and natural (N), and each host group is represented by a separate set of equations reflecting its ecological characteristics and movement patterns. An overview of the modelling framework is shown in figure 1, while the resulting baseline simulation under the default parametrization is shown in figure 2.

**Figure 2.**
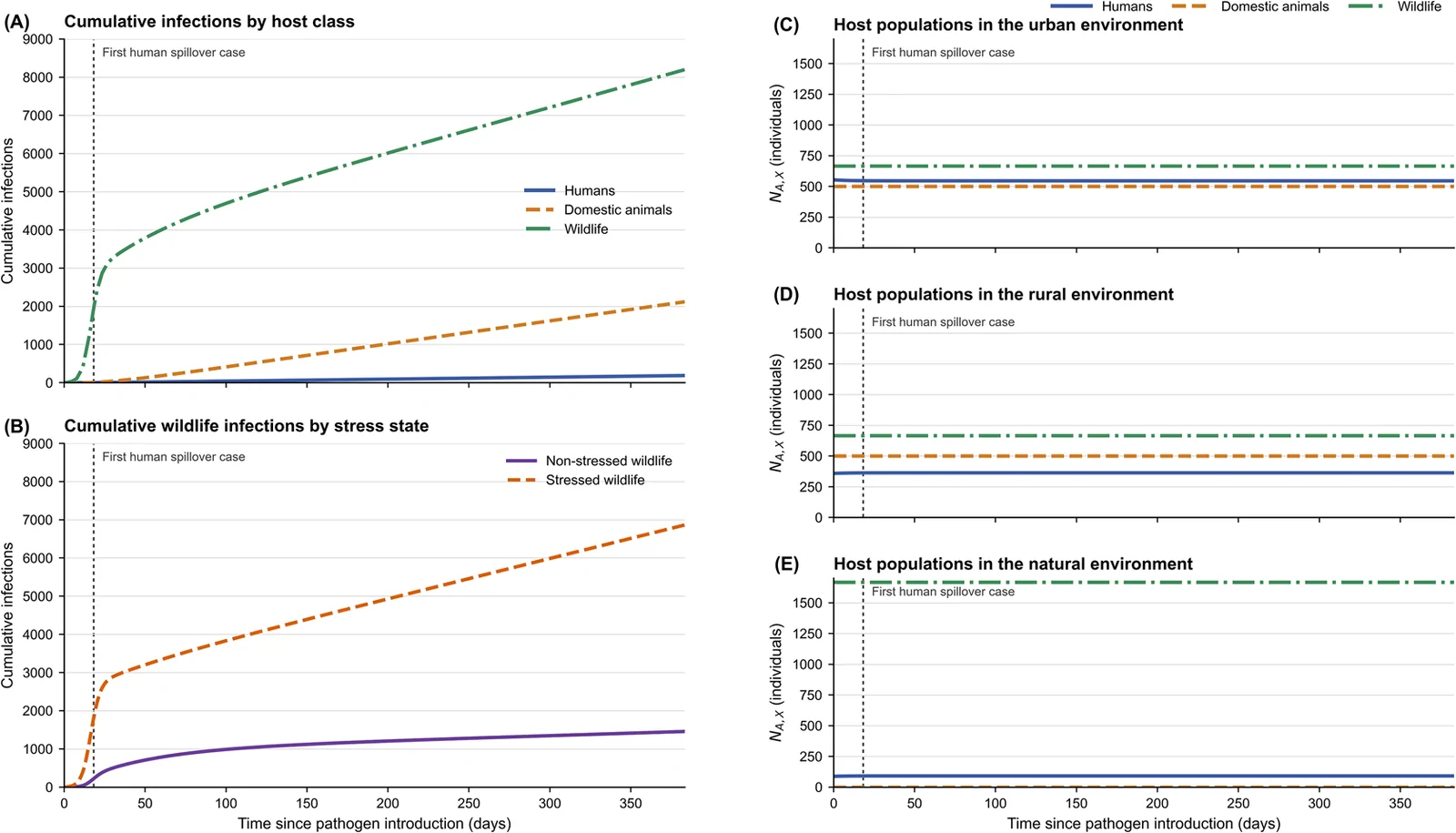
Baseline simulation of the generalized spillover model using the parameter values listed in table 1. (A) Cumulative infections in humans, domestic animals and wildlife. (B) Cumulative wildlife infections from the same simulation as in panel (A), separated into stressed and non-stressed wildlife. (C–E) Total host populations in the urban, rural and natural environments, respectively. Wildlife populations in panels (A) and (C)–(E) represent the sum of stressed and non-stressed individuals, and all host populations include both susceptible and infected individuals. The simulation begins from the pathogen-free equilibrium described in §2.3, with infection introduced through a single infected wildlife individual in the natural environment. The vertical dotted line in each panel indicates the time of the first human spillover case.

Human dynamics is described by equation (2.1). Humans are assumed to occupy and move between all three environments. Because the objective of the model is to investigate the ecological drivers of novel zoonotic spillover, humans are treated as dead-end hosts. Specifically, transmission coefficients describing infection from humans to any host are set to zero, allowing humans to become infected from domestic animals or wildlife while preventing onward human transmission:

(2.1a)S˙H,p=γHIH,p−SH,p∑k∈{D,Ws,Wns}βk→HCk,H,pIk,pNk,p+∑j∈{U,R,N}j≠p[μj→pHSH,j−μp→jHSH,p](2.1b)and I˙H,p=−γHIH,p+SH,p∑k∈{D,Ws,Wns}βk→HCk,H,pIk,pNk,p+∑j∈{U,R,N}j≠p[μj→pHIH,j−μp→jHIH,p].

In equation (2.1), susceptible humans become infected according to the force of infection generated by infectious domestic animals and wildlife occupying the same environment and recover at rate $\gamma_{H}$.

Transmission from infectious stressed wildlife occurs at the stress-scaled transmission probability,

(2.2) $$\beta_{W_{s}\to A}=r_{T}^{W_{s}}\beta_{W\to A},$$

while transmission from infectious non-stressed wildlife occurs at the baseline wildlife-to-population A transmission probability,

(2.3) $$\beta_{W_{\mathrm{ns}}\to A}=\beta_{W\to A}.$$

Similarly, transmission to stressed wildlife occurs at the stress-scaled transmission probability,

(2.4) $$\beta_{A\to W_{s}}=r_{S}^{W_{s}}\beta_{A\to W}.$$

Movement between environments is governed by the directional per capita movement rates $\mu_{X\to Y}^{A}$, where A denotes the host class, and X and Y denote the origin and destination environments, respectively. Thus, $\mu_{j\to p}^{H}$ represents movement of humans from environment j into environment p, while $\mu_{p\to j}^{H}$ represents movement from environment p to environment j.

Domestic animal dynamics is described by equation (2.5) as follows:

(2.5a)S˙D,p=γDID,p−SD,p∑k∈{D,Ws,Wns}βk→DCk,D,pIk,pNk,p+∑j∈{U,R,N}j≠p[μj→pDSD,j−μp→jDSD,p](2.5b)and I˙D,p=−γDID,p+SD,p∑k∈{D,Ws,Wns}βk→DCk,D,pIk,pNk,p+∑j∈{U,R,N}j≠p[μj→pDID,j−μp→jDID,p].

In these equations, domestic animals are represented as a single host class that may occupy urban or rural environments. In the baseline parametrization, the urban population primarily represents companion animals, whereas the rural population represents livestock. Movement between environments is included to provide a general framework for different domestic animal populations, although the baseline movement rates are zero. In equation (2.5), susceptible domestic animals become infected through contact with infectious domestic animals and wildlife occupying the same environment and recover at rate $\gamma_{D}$.

Wildlife dynamics is described by equation (2.6). We consider a generalized wildlife population representing a habitat-generalist reservoir host capable of occupying urban, rural and natural environments and moving between them. This choice is motivated by evidence that human-modified landscapes are often favourable for disturbance-tolerant, habitat-generalist species that comprise a disproportionate fraction of known zoonotic reservoir hosts [37]. Accordingly, the framework is intended to represent wildlife populations that exploit multiple habitat types and may facilitate pathogen transmission across wildlife, domestic animal and human populations. Host competence is assumed to be an inherent species- and pathogen-specific characteristic, while chronic physiological stress is modelled separately as a modifier of transmission-related processes rather than a determinant of host competence itself. Wildlife is further divided into stressed and non-stressed subpopulations to represent changes in physiology and behaviour associated with anthropogenic disturbance:

S˙Wns,p=γWIWns,p−SWns,p∑k∈{D,Ws,Wns}βk→WnsCk,Wns,pIk,pNk,p+σpWSWs,p−ρpWSWns,p(2.6a)+∑j∈{U,R,N}j≠p[μj→pWSWns,j−μp→jWSWns,p],S˙Ws,p=rRWsγWIWs,p−SWs,p∑k∈{D,Ws,Wns}βk→WsCk,Ws,pIk,pNk,p−σpWSWs,p+ρpWSWns,p(2.6b)+∑j∈{U,R,N}j≠p[μj→pWSWs,j−μp→jWSWs,p],I˙Wns,p=−γWIWns,p+SWns,p∑k∈{D,Ws,Wns}βk→WnsCk,Wns,pIk,pNk,p(2.6c)+∑j∈{U,R,N}j≠p[μj→pWIWns,j−μp→jWIWns,p]and I˙Ws,p=−rRWsγWIWs,p+SWs,p∑k∈{D,Ws,Wns}βk→WsCk,Ws,pIk,pNk,p(2.6d)+∑j∈{U,R,N}j≠p[μj→pWIWs,j−μp→jWIWs,p].

Equation (2.6) incorporates susceptible wildlife transition to non-stressed and stressed states at environment-specific rates $\sigma_{p}$ and $\rho_{p}$, while movement between environments is represented by movement terms similar to those used for humans. We assume that infected individuals retain the stress state they occupied at the time of infection until recovery.

Disease transmission in equations (2.1), (2.5) and (2.6) depends on the contact rate between host populations (H,D and W) and the probability of infection given contact. Human-to-human contact is assumed to be frequency dependent, with a constant per capita contact rate, whereas all other host pairs are assumed to follow symmetric density-dependent contact rates,

(2.7) $$C_{i,j,p}=c_{i,j}(N_{j,p}{)}^{0.5},$$

where $c_{i,j}$ is a population-pair-specific coefficient and $N_{j,p}$ is the population size of host population j in environment p. Equation (2.7) provides a simple representation of density-dependent contact that is consistent with empirical evidence [38]. The baseline contact coefficients were selected to produce biologically plausible contact rates across the simulated population sizes, guided by the contact-density relationship described by Borremans *et al.* [38]. In assigning the baseline coefficients, we assumed that contact rates were highest within host species, followed by contacts between humans and domestic animals, then between wildlife and domestic animals, with direct human–wildlife contacts occurring least frequently. We also examined the resulting contact rates across the baseline population sizes to ensure that they produced a reasonable illustrative scenario without implausibly low or high contact frequencies. Because the model is not parametrized for a particular host system, these values are not intended as empirical estimates and are varied over broad ranges in the global sensitivity analysis.

The three environments are represented as interconnected patches, each containing susceptible and infected humans, domestic animals, stressed wildlife and non-stressed wildlife, resulting in a total of 24 state variables. Populations are assumed to be well mixed within each environment, and transmission occurs only among individuals occupying the same environment. Environmental transmission is not modelled explicitly, but indirect routes such as food contamination are assumed to be reflected in the effective contact rates.

### 2.2. Stress formulation

In this framework, we assume that wildlife individuals become chronically stressed and recover from chronic stress at rates determined by environmental conditions. Here, chronic stress refers to a sustained physiological state arising from persistent or repeated stressors, or from stressors whose physiological effects persist beyond the immediate response. The onset and recovery of chronic stress for wildlife are specified separately for each patch type, as illustrated in figure 3A, but empirical data to inform these rates remain extremely limited.

**Figure 3.**
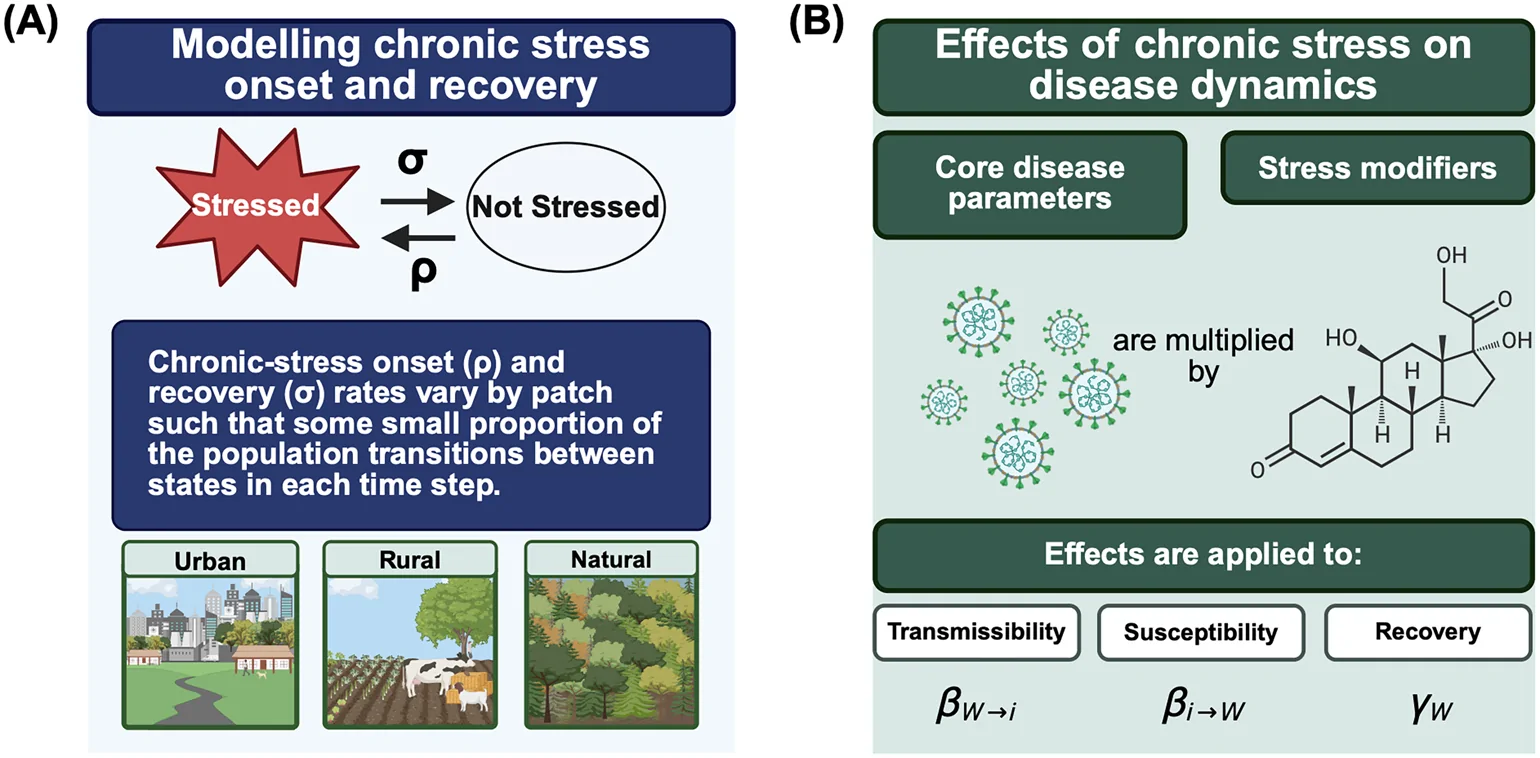
Conceptual representation of chronic stress processes in the model. (A) Implementation of stress onset and recovery in the model, where wildlife becomes chronically stressed and recover from chronic stress at environment-specific rates. (B) Physiological pathways represented in the model, in which chronic stress modifies epidemiological parameters by increasing susceptibility and transmissibility and reducing recovery rates through multiplicative stress modifiers, thereby extending infectious periods and enhancing transmission potential across host interfaces (Created in BioRender: Stafford, E. (https://BioRender.com/qxcja5o) is licensed under CC BY 4.0.).

Empirical evidence shows that chronic stress can increase susceptibility, prolong infectiousness and increase transmissibility [39,40]. These mechanisms, summarized conceptually in figure 3B, are represented in the model through multiplicative scaling factors applied directly to these core epidemiological parameters. Specifically, stress affects three epidemiological parameters for wildlife. First, it increases the probability that an infectious wildlife individual transmits the pathogen during a contact (transmissibility; $\beta_{W\to i}$), reflecting increased viral shedding under stress. Second, it increases the probability that a wildlife individual becomes infected when contacting any infectious individual (susceptibility; $\beta_{i\to W}$), reflecting stress-related immune suppression. Finally, it lowers the per capita recovery rate ($\gamma_{W}$), thereby lengthening the infectious period. This implementation parallels the scalar multiplier approach of Owen *et al.* [27] for West Nile virus, but generalizes it to a multi-species, multi-environment framework in which stress dynamically modulates cross-species spillover risk. The model does not distinguish between specific stressors, instead representing their combined effects through changes in susceptibility, transmissibility and recovery to preserve generality across ecological and epidemiological contexts. In practice, such sustained physiological stress may arise from diverse processes, including persistent anthropogenic disturbance, seasonal food limitation, prolonged reproductive demands or lasting physiological effects of extreme environmental events, each of which may contribute differently to pathogen transmission in particular host–pathogen systems.

Chronic stress may also influence spillover risk through behavioural changes, such as altered movement between environments or changes in contact rates. These effects are not modelled explicitly, as their magnitude and direction vary widely across species and contexts. However, their influence can be inferred from the effects of varying the parameters governing movement and contact rates, which capture how behavioural shifts may modulate transmission dynamics.

In our baseline-scenario parametrization (table 1), stress onset and recovery depend on the balance between resource availability and anthropogenic disturbance. We deliberately consider a habitat-generalist wildlife population for which human-modified rural environments can provide a resource-rich refuge relative to degraded or resource-limited natural habitat. Accordingly, rural patches are modelled as low-stress environments with no chronic stress onset and a relatively high rate of stress recovery, reflecting access to abundant anthropogenic or agricultural food resources with lower levels of disturbance than in urban environments. Urban patches are assumed to contain abundant resources but experience a high rate of chronic stress onset owing to intense anthropogenic disturbance, with no recovery from chronic stress. Natural patches are assigned a higher rate of chronic stress onset than urban patches and, in the baseline scenario, no stress recovery, representing natural environments in which reduced habitat extent, low productivity or seasonal food limitation constrains resource availability. This parametrization is therefore not intended to imply that rural environments are generally less stressful for wildlife than natural environments, but rather represents an illustrative ecological scenario relevant to habitat-generalist species that can benefit from resources associated with human-modified landscapes. This baseline applies to wildlife systems in which natural areas no longer support resource requirements at levels sufficient to offset physiological stress, a condition increasingly common under habitat loss and environmental change [41]. These assumptions are relaxed in the global sensitivity analysis, where stress onset and recovery rates are allowed to vary widely and independently across environments to account for ecological and physiological uncertainty.

**Table 1.** Model parameters, baseline values and ranges used in the global sensitivity analysis. Host-indexed vectors follow the order (H,D,W) for humans, domestic animals and wildlife, respectively. Environment-indexed vectors follow the order (U,R,N) for urban, rural and natural environments, respectively. Directional movement vectors contain the off-diagonal movement rates in the order (U→R,U→N,R→U,R→N,N→U,N→R). Diagonal entries of the corresponding movement matrices are calculated as the negative sum of the outgoing rates and are not independently varied parameters.

parameter	symbol	description and ordering	baseline value	sensitivity range	units
total population	$N_{A}$	total population size of host class A, calculated as the sum of its environment-specific populations.	$N_{H}=1000$ $N_{D}=1000$ $N_{W}=3000$	derived from the environment-specific population sizes	individuals
initial population by environment	$N_{A,X}$	initial population of host class A across environments X, ordered as (U,R,N).	$N_{H,X}=(900,100,0)$ $N_{D,X}=(500,500,0)$ $N_{W,X}=(1000,1000,1000)$	$N_{H,X}:([450,1350],[75,225],[0,100])$ $N_{D,X}:([250,750],[350,1050],[0,100])$ $N_{W,X}:(1000,1000,[500,5000])$	individuals
human–human contact rate	$C_{H,H}$	frequency-dependent contact rate between humans	12	not varied	days${}^{-1}$
density-dependent contact coefficient	$c_{A,B}^{X}$	contact coefficients ordered as $(c_{H,D},c_{H,W},c_{D,D}$, $c_{D,W},c_{W,W})$. Reciprocal between-host coefficients are equal. The same baseline coefficients are used in each environment, X.	(0.05,0.005,0.15, 0.015,0.15)	$c_{H,D}^{X}:[0.005,0.1]$, $c_{H,W}^{X}:[0.005,0.01]$, $c_{D,D}^{X}:[0.005,0.2]$, $c_{D,W}^{X}:[0.005,0.01]$, $c_{W,W}^{X}:[0.015,0.2]$	individuals${}^{-1/2}$ days${}^{-1}$
transmission probability per contact	$\beta_{A\to B}$	transmission probabilities ordered as (D→H, W→H, D→D,W→D,D→W, W→W). Transmission from humans is set to zero.	(0.05,0.025, 0.5, 0.05,0.05,0.5)	D→H:[0.01,0.1] W→H:[0.01,0.1] D→D:[0.05,0.5] W→D:[0.05,0.1] D→W:[0.05,0.1] W→W:[0.05,0.5]	dimensionless
recovery rate	$\gamma_{A}$	per capita recovery rates in host ordered as (H,D,W).	(1/5,1/10,1/14)	$\gamma_{H}$: not varied $\gamma_{D}:[1/30,1/5]$ $\gamma_{W}:[1/30,1/5]$	days${}^{-1}$
human movement rates	$\mu_{X\to Y}^{H}$	off-diagonal directional movement rates ordered as specified in the table caption.	(0.05,0,0.05, 0.05,0.1,0.1)	each directional rate was varied independently over [0,0.25].	days${}^{-1}$
domestic animal movement rates	$\mu_{X\to Y}^{D}$	off-diagonal directional movement rates ordered as specified in the table caption.	(0,0,0,0,0,0)	each directional rate was varied independently over [0,0.25].	days${}^{-1}$
wildlife movement rates	$\mu_{X\to Y}^{W}$	off-diagonal directional movement rates ordered as specified in the table caption.	(0.25,0.25,0.25, 0.25,0.1,0.1)	each directional rate was varied independently over [0,0.5].	days${}^{-1}$
stress onset rate	$\rho_{X}$	environment-specific rates at which non-stressed wildlife becomes chronically stressed, ordered as (U,R,N).	(0.1,0,0.3)	each environment-specific rate was varied independently over [0,1].	days${}^{-1}$
stress recovery rate	$\sigma_{X}$	environment-specific rates at which stressed wildlife return to the non-stressed state, ordered as (U,R,N).	(0,0.1,0)	each environment-specific rate was varied independently over [0,1].	days${}^{-1}$
initial infected, non-stressed wildlife	$I_{0,\mathrm{ns}}^{W}$	initial infected, non-stressed wildlife across environments ordered as (U,R,N).	(0,0,1)	not varied	individuals
initial infected, stressed wildlife	$I_{0,s}^{W}$	initial infected, stressed wildlife across environments ordered as (U,R,N).	(0,0,0)	not varied	individuals
initial stressed wildlife	$S_{0,s}^{W}$	initial susceptible, stressed wildlife across environments ordered as (U,R,N).	(0,0,0)	not varied	individuals
susceptibility scaling	$r_{S}^{W_{s}}$	multiplicative effect of chronic stress on wildlife susceptibility to infection.	2	[1,4]	dimensionless
transmissibility scaling	$r_{T}^{W_{s}}$	multiplicative effect of chronic stress on transmission from infected wildlife	2	[1,4]	dimensionless
recovery scaling	$r_{R}^{W_{s}}$	multiplicative effect of chronic stress on the recovery rate of infected wildlife	0.05	[0.05,1]	dimensionless

### 2.3. Simulation initialization

To generate realistic initial conditions, we first ran the system without infection—retaining host movement and wildlife stress dynamics—until it reached equilibrium. This produced stable spatial distributions and stress states in a pathogen-free setting. Once the pathogen-free system reached equilibrium, we introduced a single infected wildlife individual into the natural patch to seed pathogen invasion. This system state—comprising stressed and unstressed wildlife, susceptible individuals of all species across environments and one infected wildlife individual—was used as the initial condition for all simulation runs, with all species assumed susceptible to the pathogen. The resulting baseline simulation, shown in figure 2, illustrates the subsequent infection dynamics and redistribution of host populations across the three environments under the default parametrization. This represents the emergence of a novel zoonotic pathogen capable of spreading between wildlife, domestic animals and humans.

### 2.4. Sensitivity analyses

We conducted both local and global sensitivity analyses to quantify the influence of wildlife stress on spillover outcomes and to identify parameters that are most influential for model behaviour and calibration in case-study applications. These analyses build on the baseline simulation shown in figure 2, with the local analysis quantifying perturbations around this reference scenario and the global analysis exploring a much broader parameter space. Local sensitivity analysis quantifies how small perturbations around a baseline parameter set affects model output, reflecting system responsiveness under current or assumed conditions. As such, it is useful for identifying parameters that may be most effectively targeted by mitigation measures, such as interventions in domestic animal health or habitat quality. By contrast, total-order ($S_{T}$) Sobol’ indices from global sensitivity analysis capture interaction effects and system behaviour across a wide range of ecological and epidemiological conditions, which may not correspond to any single realized scenario. Consequently, the global analysis highlights parameters that consistently influence outcomes across the parameter space, revealing system-level drivers and potential sources of instability or emergence. Together, these approaches distinguish between factors that are locally important under specific conditions and those that are globally influential across plausible system configurations, providing a more comprehensive understanding of spillover dynamics.

The sensitivity analysis varied the epidemiological, movement, population, contact and stress-related parameters as listed in table 1. This includes epidemiological, movement, demographic and stress-related parameters across all host populations, as well as the stress-scaling parameters governing how chronic stress alters susceptibility, transmissibility and recovery in wildlife.

For the sensitivity analyses, we focus on how different parametrizations affect two quantities of interest (QOI). The first is the time to the first spillover event into humans, which can occur through contact with either infected wildlife or domestic animals; this serves as a proxy for a public health notification event, marking the point at which a new zoonotic pathogen would first be detected in humans. After this initial spillover event, the model is run for an additional year of simulation time, during which we measure the spillover incidence rate, defined as the per-population number of human infections per 100 000 individuals per year. These QOIs thus describe both the timing and intensity of zoonotic spillover events. A summary of the parameters of interest (POI) is listed in table 1, and the QOIs used in our analyses are listed in table 2.

**Table 2.** Quantities of interest (QOI) used in local and global sensitivity analyses.

QOI	definition / units
time to first spillover event	time from simulation start until the first human infection occurs (days).
spillover incidence per year	number of human infections per 100 000 occurring within a one-year window after the first human infection (events ⋅ year${}^{-1}$).

To perform the sensitivity analyses, we define a baseline system representing one ecological scenario relevant to zoonotic spillover. This baseline provides the reference conditions for the local sensitivity analyses. The model is intended as a generalized framework rather than a representation of a particular wildlife species or ecosystem. Consequently, equal initial wildlife populations were assigned to the urban, rural and natural environments as a neutral starting point because no species-specific abundance or habitat distribution was assumed. In this set-up, humans primarily occupy urban areas with limited movement into rural and natural environments; wildlife uses all environments; and domestic animals remain mostly within their initial locations. These movement assumptions determine contact patterns and exposure conditions and are therefore central to the local analysis. In the global sensitivity analysis, we expand beyond this baseline to capture a broader range of ecological and behavioural systems.

#### 2.4.1. Local sensitivity analysis

We first perform a local sensitivity analysis to examine how small perturbations in individual parameters around the baseline (table 1) affect our QOIs. This type of analysis is valuable not only for its low computational cost but also for the insights it offers into parameter estimations. Parameters that exhibit high local sensitivity highlight where efforts should be focused on data collection. In the context of our model, local sensitivity analysis can provide useful insights into the effectiveness of mitigation strategies targeting specific dynamics, such as reducing contact rates or increasing recovery rates. Local sensitivity indices are reported as elasticities around the baseline; the sign indicates direction of change in the QOI (positive increases, negative decreases) for a small increase in the parameter.

We calculate the local sensitivity of our QOIs to our POIs using standard sensitivity indices [42]. These indices are computed by perturbing each POI slightly around its baseline value and evaluating the resulting change in the QOI; this finite-difference approximation provides an estimate of the derivative dq/dp. The sensitivity index is then defined as

(2.8) $$S_{p}^{q}=\left(\frac{\text{d}q}{\text{d}p}\right)\cdot \left(\frac{p^{*}}{q^{*}}\right),$$

where q is the QOI, p is the POI and the asterisks denote the baseline values. The expression on the right-hand side consists of two multiplicative factors: the local rate of change (dq/dp) and a scaling factor that non-dimensionalizes the derivative $\left(p^{*}/q^{*}\right)$. This scaling removes units and normalizes the magnitude of the sensitivity, allowing indices for different parameters—potentially measured in different units or on different scales—to be compared directly.

#### 2.4.2. Global sensitivity analysis

Global sensitivity analysis, though much more computationally expensive, provides a more comprehensive evaluation of how changes in POIs affect QOIs. Unlike in local sensitivity analysis, all parameters are varied simultaneously, which enables us to account for nonlinearities in the model and interactions between parameters. For the global analysis, we employ variance-based Sobol’ sensitivity indices [42,43] to quantify parameter influence. Two complementary measures are reported: the first-order index ($S_{1}$) and the total-order index ($S_{T}$).

The first-order index represents the main (additive) effect of a parameter when considered individually while averaging over uncertainty in all other parameters, reflecting how much of the output variance can be attributed to that parameter alone. Unlike local sensitivity indices, which quantify the response of an output to small perturbations of a parameter around a fixed baseline, first-order Sobol’ indices characterize parameter influence across the full parameter space. The formula for the first-order contribution for a parameter $p_{i}$ on an output q is given by

(2.9) $$S_{1i}=\frac{V_{i}(q)}{V[q]},$$

such that

(2.10)Vi(q)=V[E(q|pi)],(2.11)Vij(q)=V[E(q|pipj)]−Vi(q)−Vj(q)(2.12)and V[q]=∑iVi(q)+∑i<jVij(q)+⋯+V1,2,…,N.

The total-order index ($S_{T}$) captures the full contribution of a parameter, including all higher-order interactions with other parameters (i.e. $S_{i}$, $S_{ij}$, $S_{i\ldots j\ldots k}$). The formula for the $S_{T}$ index of parameter i is given by

(2.13) $$S_{Ti}=S_{i}+\sum_{j\neq i}S_{ij}+\sum_{j,k\neq i}S_{ijk}+\cdots +S_{i\ldots N}=1-\frac{V[E(q|p_{∼i})]}{V[q]},$$

where $p_{∼i}$ indicates the set of all parameters except i [44,45].

The gap $S_{T}-S_{1}$ indicates interaction strength: values near zero imply predominantly additive effects, whereas larger gaps signal interaction-dominated influence.

Sobol’ indices were estimated using a Saltelli sampling design over independent priors for each of the parameters. Uncertainty in indices is shown as 95% confidence intervals, computed by resampling the Sobol’ sample as implemented in SALib [46,47].

## 3. Results

To characterize the dominant processes shaping pathogen transmission across hosts distributed across different environments, we evaluated model sensitivity to key epidemiological and ecological parameters. Spillover risk was quantified using two outcome QOIs: (i) the incidence of spillover events during the year following the first event (per 100 000 humans) and (ii) the time to the first spillover event. We then applied both local and global sensitivity analyses to determine how variation in each parameter, in the set of POIs, influenced these outcomes.

Figure 4 shows the 10 most influential parameters affecting the incidence of spillover events and time to first spillover event, for both global and local sensitivity analysis. On the basis of these findings, we advance two main claims: (i) reducing chronic stress in wildlife populations lowers spillover risk, and (ii) reducing spillover risk can be achieved through limiting the potential for amplification effects from domestic animals. In the following sections, we present the evidence supporting each claim and discuss its broader ecological and management implications. Complete results for all parameters are listed in tables S2– S4 in the supplementary material.

**Figure 4.**
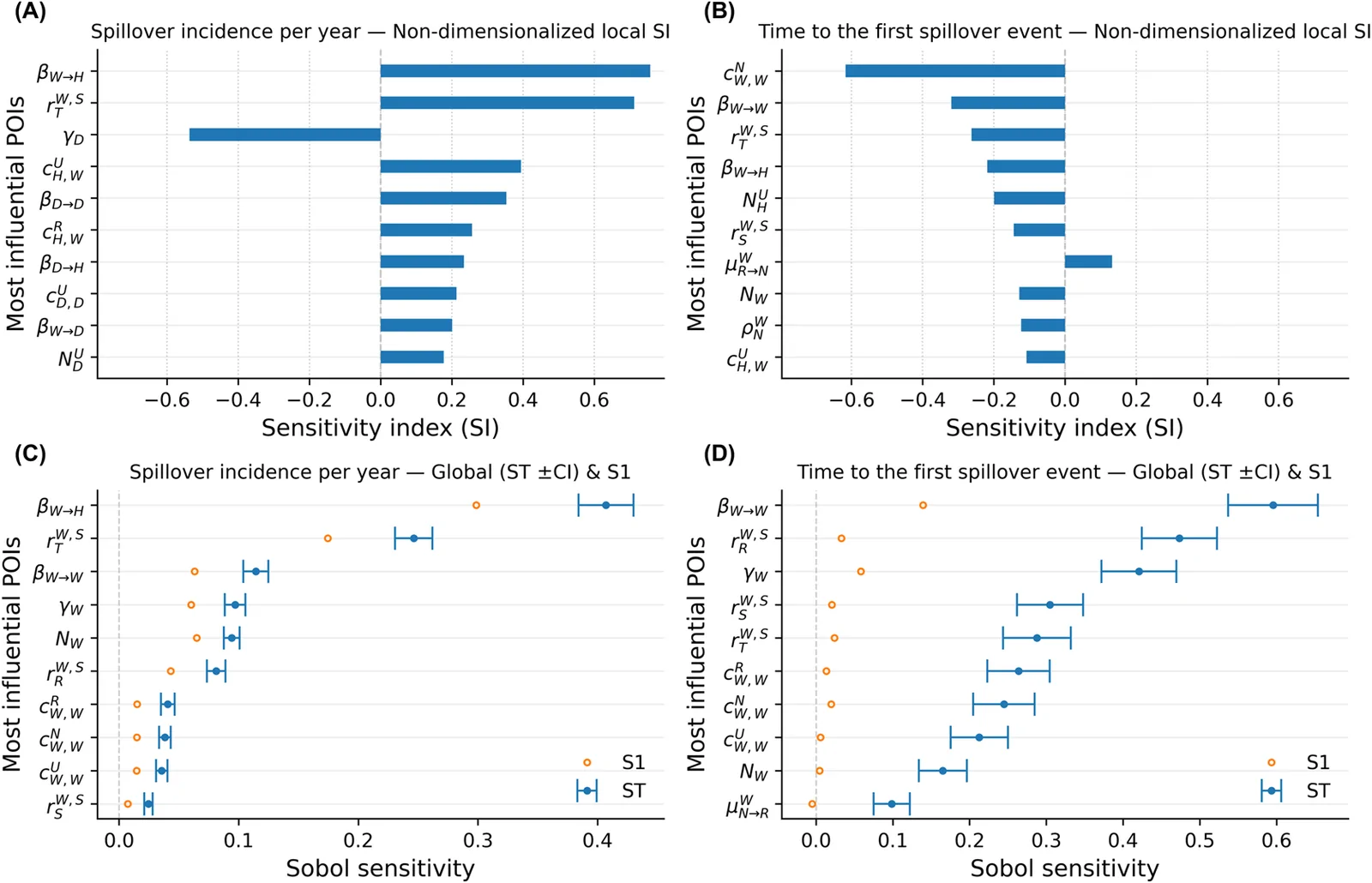
Key drivers of zoonotic spillover identified by sensitivity analyses. This figure shows the 10 most influential parameters for each outcome and metric. Across panels (A)–(D), 23 separate parameters were identified as the most influential. (A) and (B) Local sensitivity indices for (A) per-population incidence of spillover events per year and (B) time to first spillover event. Bars show the 10 POI with the highest indices. (C) and (D) Global sensitivity indices for (C) per-population incidence and (D) time to first spillover event. Filled circles represent total-effect ($S_{T}$) indices with 95% confidence intervals (whiskers); open circles represent first-order ($S_{1}$) indices. In these panels, small negative estimates are treated as approximately zero (estimation noise). The differences between first-order ($S_{1}$) and total-effect ($S_{T}$) indices for several parameters were large, indicating interaction and compounding effects.

### 3.1. Reducing chronic stress in wildlife populations decreases spillover risk

Chronic physiological stress in wildlife emerged as an important modifier of spillover dynamics across both local and global sensitivity analyses (figure 4). Stress scalings on wildlife transmissibility, recovery and susceptibility ($r_{T}^{W,S}$, $r_{R}^{W,S}$, $r_{S}^{W,S}$) ranked among the most influential parameters (figure 4), indicating that chronic physiological stress in wildlife amplifies spillover risk to humans. Specifically, the transmissibility and susceptibility scalings were significant in both outcomes and analyses and the recovery scaling was influential globally. The rate of chronic stress onset in natural environments ($\rho_{N}^{W}$) was also locally important for time to first spillover, suggesting that stress-promoting conditions in the natural environment can accelerate emergence in our baseline scenario. The influence of stress effects in both the local and global analyses shows that chronic stress holds system-wide influence on spillover dynamics.

These stress effects are superimposed on a set of core epidemiological processes—namely, wildlife transmission probabilities, recovery and population size—that govern disease dynamics independently of behavioural or stress-related mechanisms. Across analyses, these baseline disease parameters were consistently among the strongest drivers of both spillover incidence and time to first spillover (figure 4). For spillover incidence in humans, the transmission probability per infectious contact from wildlife to humans ($\beta_{W\to H}$) was the most important driver in both the local and global analyses. The transmission probability from wildlife to domestic animals ($\beta_{W\to D}$) was additionally highlighted in the local results, while transmission among wildlife ($\beta_{W\to W}$), wildlife recovery rate ($\gamma_{W}$) and total wildlife population size ($N_{W}$) were prominent in the global analyses. For the time to the first spillover event, transmission among wildlife and contact rates among wildlife ($c_{W}^{N}$, $c_{W}^{R}$, $c_{W}^{U}$) were among the most influential parameters across analyses, and the wildlife recovery rate ranked third in the global results. These findings highlight the role of wildlife in shaping spillover potential. In addition to these core epidemiological processes, with or without stress modifiers, other behavioural mechanisms may further influence spillover dynamics and may themselves be affected by chronic stress.

Chronic stress can also alter wildlife behaviour. These stress-related shifts in wildlife movement and contact structure can substantially influence zoonotic spillover dynamics by reshaping opportunities for cross-species transmission. Although we do not model stress-related changes in behaviour directly, we do include behavioural parameters (i.e. $c_{H,W}^{U}$; $c_{H,W}^{R}$; $c_{W}^{N}$; $\mu_{R\to N}^{W}$ and $\mu_{N\to R}^{W}$) in the sensitivity analysis and can infer what effects behavioural changes may lead to in the QOIs. In the local analysis, higher human–wildlife contact in urban and rural environments ($c_{H,W}^{U}$, $c_{H,W}^{R}$) increased spillover incidence, and contact among wildlife in natural habitats ($c_{W}^{N}$) strongly affected time to first spillover. Moreover, contact among wildlife in all habitats strongly affected both spillover incidence and the time to the first spillover in the global analysis. Movement between rural and natural ($\mu_{R\to N}^{W}$ and $\mu_{N\to R}^{W}$) patches also shaped timing, with greater exchange generally lengthening time to first spillover.

To compare the relative importance of these mechanisms, we grouped the wildlife parameters into three categories: core epidemiological processes (transmission probabilities, recovery and population size), behavioural processes (contact and movement) and stress-related processes (stress onset, stress recovery and stress-induced modifiers of susceptibility, transmissibility and recovery). For each category, we calculated the mean total-effect Sobol’ index (${\bar{S}}_{T}$) across all parameters in the respective categories, based on the global sensitivity analysis for each outcome. These averaged indices quantify the average proportion of model output variance attributable to parameters in each group, accounting for both direct and interaction effects. This allows for a direct comparison of the relative contribution of the studied epidemiological, physiological and behavioural processes to spillover risk by determining the ratio of these effects across the three parameter categories (figure 5A,B). Across both outcomes, core epidemiological processes and stress-related parameters explained more variance than behavioural factors. For spillover incidence, core epidemiological processes were most influential (approx. 8.4× behavioural) and stress modifiers more than twice as strong as behavioural parameters (approx. 2.8×). For time to first spillover, core epidemiological processes still dominated (approx. 2.4× behavioural), but the effect was less pronounced, with stress-related parameters contributing moderately (approx. 1.5× behavioural). All ratios are based on normalized ${\bar{S}}_{T}$ values to allow comparison across outcome measures, while the parameter groupings are illustrated in figure 5C.

**Figure 5.**
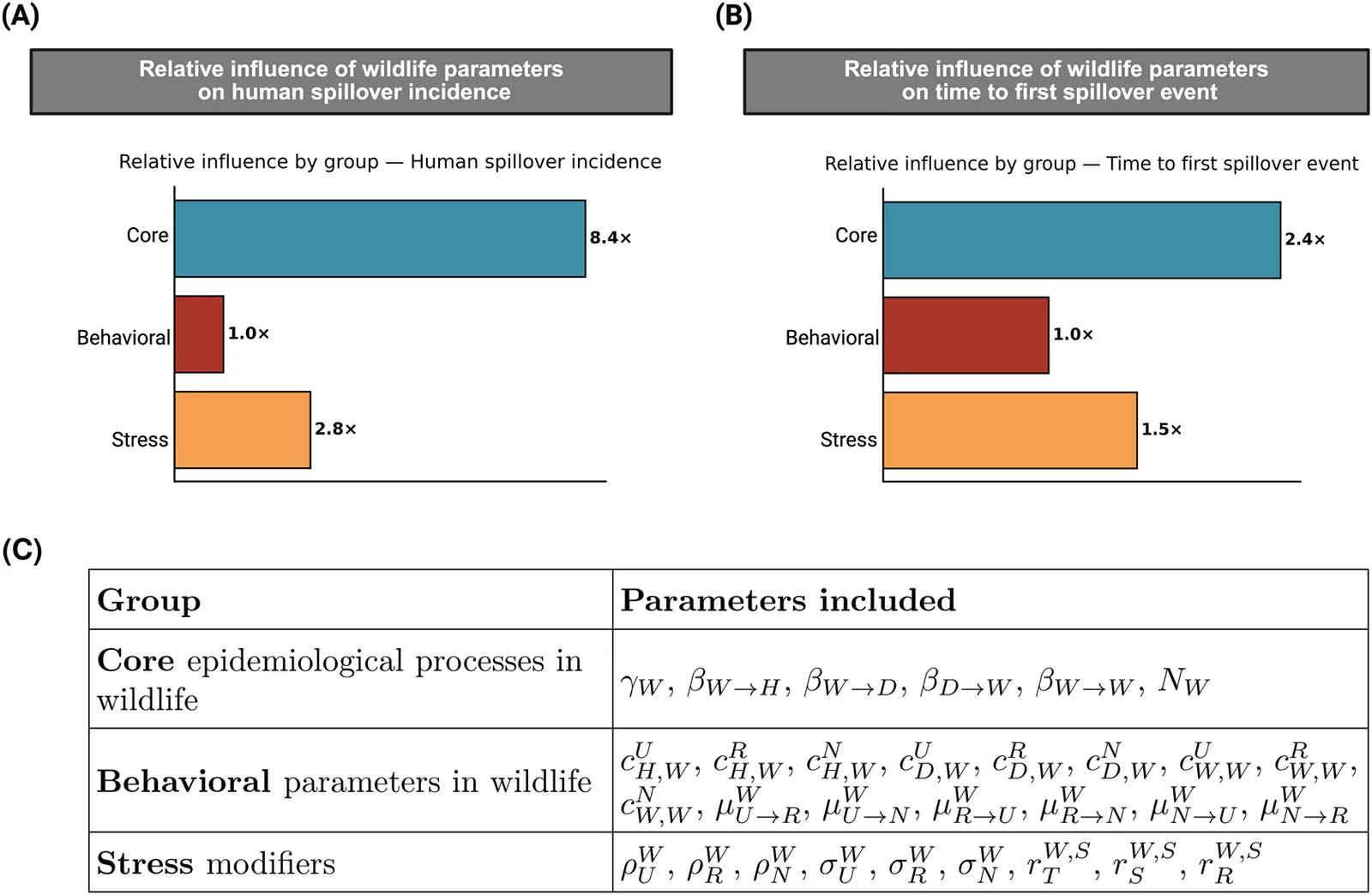
Relative influence of wildlife parameter groups on spillover outcomes. (A)–(B) Comparison of wildlife parameter groups. Mean total-effect Sobol’ indices (${\bar{S}}_{T}$) were calculated by averaging the total-effect Sobol’ indices across all parameters within each group. Results are shown for the global sensitivity analysis of each QOI. (C) Wildlife parameter grouping used in the analysis. Core epidemiological processes comprise the wildlife transmission probabilities, recovery rate and population size; behavioural processes comprise the wildlife contact and movement rates; and stress-related processes comprise the stress onset and recovery rates together with the stress-induced modifiers of susceptibility, transmissibility and recovery.

Chronic stress, through its effects on susceptibility, transmissibility and recovery, exerts an influence on spillover-related outcomes comparable to that of core epidemiological processes. Our analyses indicate that these two factors are the most influential in this context, explaining more variance than behavioural parameters. This emphasizes the need for empirical data on physiological stress indicators—such as cortisol or other biomarkers—when parametrizing spillover models. At the same time, behavioural processes remain integral: movement, contact structure and environment-specific stress dynamics determine which hosts experience stress and where cross-species encounters occur. Behavioural processes therefore define the ecological context—shaped by habitat state and land-use change (e.g. [48])—within which stress-related mechanisms operate, even though behaviour explains less variance than physiological parameters in the Sobol’ analysis.

### 3.2. Reducing spillover risk by limiting domestic animal amplification

Within the local analysis, several domestic parameters were highly influential (figure 4). The domestic animal recovery rate ($\gamma_{D}$) ranked third overall, with faster recovery reducing spillover incidence, while the probability of transmission per infectious contact among domestic animals ($\beta_{D\to D}$) and from domestic animals to humans ($\beta_{D\to H}$) were also important. Other top parameters included contact rates among domestic animals in urban environments ($c_{D,D}^{U}$) and the population size of domestic animals in the urban interface ($N_{D}^{U}$). Taken together, these results are consistent with a domestic amplification mechanism, in which high domestic animal densities, transmission from wildlife to domestic animals and the effects of poor biosecurity surrounding domestic animals (e.g. slow recovery, high per-contact transmission probability to and from domestic animal populations) increase infection pressure at human–animal interfaces, elevating spillover risk to humans (figure 6A; supported by Jones *et al.* [49] and Sikkema & Koopmans [50]).

**Figure 6.**
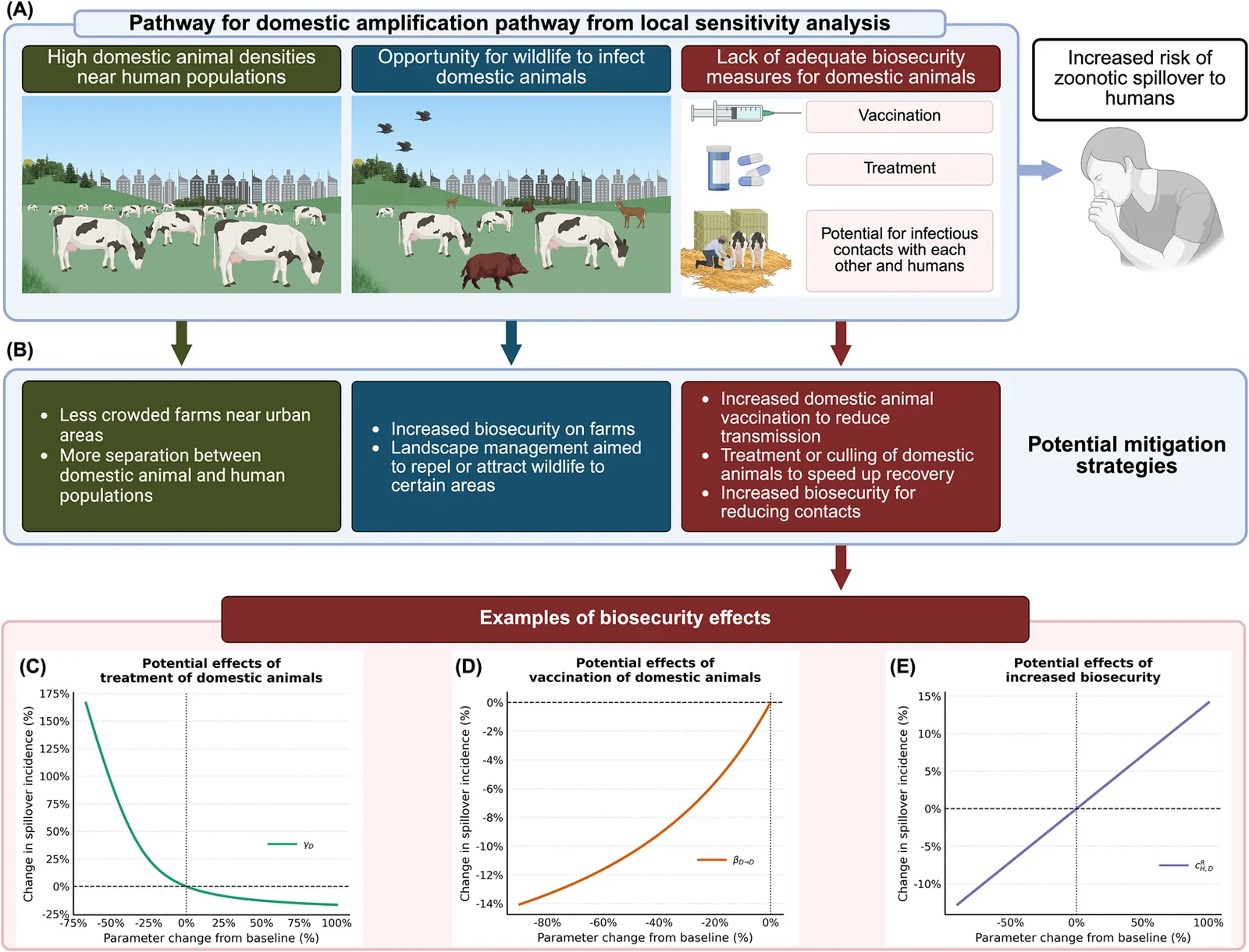
Domestic amplification of zoonotic spillover risk. (A) Conceptual schematic summarizing the local sensitivity analysis: infection introduced from wildlife spreads within dense, connected domestic populations and elevates infection pressure at human–animal interfaces. (B) Examples of mitigation strategies for reducing the parameters corresponding to the effects in panel (A). (C)–(E) Sensitivity of predicted human spillover incidence during the year after the first spillover event to key parameters concerning biosecurity in domestic animal populations: (C) recovery rate in domestic animals ($\gamma_{D}$), (D) within-domestic per-contact transmission probability ($\beta_{D}\to D$) and (E) human–domestic contact in agricultural interfaces ($c_{H,D}^{R}$). Lines show per cent change in spillover incidence relative to baseline as each parameter varies across its plausible range. (Created in BioRender: Stafford, E. (2026) https://BioRender.com/wi320g2).

These results indicate that mitigation strategies targeting domestic animals, such as those mentioned in figure 6B, are likely to be effective near the baseline regime explored by the local analysis. Moreover, As shown in figure 6C–E, incremental improvements in biosecurity—through faster recovery (treatment), lower within-domestic per-infectious-contact transmission probability (vaccination) or reduced human–domestic contact—translate into reductions in human spillover incidence. Accordingly, strengthening health management and improving biosecurity represent practical, actionable levers to mitigate spillover risk.

## 4. Conclusions

This study advances current understanding of zoonotic disease emergence by explicitly incorporating chronic stress as a dynamic modifier of transmission parameters within a generalizable multi-host, multi-environment modelling framework. Through a combination of local and global sensitivity analyses, we identified wildlife chronic stress, cross-species contact structure and domestic amplification processes as key determinants of spillover risk. Stress-related parameters were consistently influential across both outcome measures (QOI), while recovery and transmission dynamics within domestic animals strongly modulated spillover incidence at the human–animal interface. The results demonstrate that even small increases in chronic stress in wildlife can markedly accelerate spillover events, while within domestic animal populations recovery and transmission dynamics strongly modulate the extent of amplification along the wildlife–domestic–human interface.

Our findings provide quantitative support for empirical evidence linking environmental change, chronic stress and spillover risk. Landscape-scale disturbances such as habitat loss, agricultural expansion and urbanization are known to greatly alter the physiology, ecology and movement of wildlife hosts, driving these animals into closer proximity with humans and domestic species [51,52]. Recent studies further indicate that stress from environmental change [53], particularly when chronic, can impair host immune defences and increase infection susceptibility and pathogen shedding in wildlife populations [24]. Anthropogenic factors not traditionally associated with disease ecology—such as noise pollution—are also emerging as modifiers of immune function and infection outcomes [54]. In addition to these physiological effects, our global analyses indicate that stress-induced changes in behaviour—particularly increased contact and movement among wildlife across environments (as a response to ecological disturbance)—can further amplify spillover potential. Together, these findings underscore the growing recognition that stress physiology operates as an important driver of pathogen transmission dynamics.

The importance of domestic animals in our analyses supports the long-standing hypothesis of domestic amplification at agricultural interfaces, whereby high-density and highly connected domestic populations facilitate pathogen flow from wildlife to humans [55,56]. This amplification effect was strongest under near-baseline (local) conditions, suggesting that it represents a context-dependent mechanism operating most strongly in realistic ecological settings. This pattern is consistent with recent evidence that companion animals and peri-domestic livestock act as bridging hosts in both rural and urban environments [57]. Within this framework, interventions that improve animal health—through vaccination, veterinary care or improved husbandry—reduce both within-species transmission and the likelihood of onward transmission to humans.

At the same time, the strong influence of stress-modulated disease dynamics in wildlife highlights the need to consider environmental and behavioural factors in spillover prevention. The model results suggest that interventions aimed at reducing chronic stress in wildlife populations—through habitat protection, restoration of resource continuity and mitigation of disturbance (including noise, light and land conversion)—can indirectly lower cross-species transmission risk. In urban and peri-urban contexts, where human–wildlife and wildlife–domestic contact rates are highest, policies that manage waste, limit foraging opportunities for wildlife and guide pet management practices may be particularly effective. Such measures complement, rather than replace, traditional veterinary and public health strategies.

By identifying the most sensitive parameters influencing spillover, this framework also provides a quantitative basis for prioritizing field data collection. Key parameters such as stress multipliers on susceptibility and transmissibility, wildlife–domestic contact rates and domestic recovery rates can be estimated through integrated ecological and veterinary surveillance. In particular, integrated ecological and veterinary surveillance that combines physiological biomarkers, such as glucocorticoids measured from faeces, hair, feathers or other non-invasive samples [58–60], with immune markers, body condition, infection status, habitat quality, resource availability, anthropogenic disturbance and movement patterns could improve estimation of stress-related parameters. Such data would not only refine model calibration but also help identify physiological and ecological indicators that could be incorporated into wildlife biosurveillance, enabling model-driven assessment of where and when spillover risk is most acute. Such surveillance should focus on measurable physiological and ecological indicators rather than attempting to classify wildlife as ‘healthy’ or ‘unhealthy’, recognizing that chronic stress responses and their epidemiological consequences are inherently species- and context-dependent.

While the present model does not include environmental transmission (e.g. pathogens latent in soil, water or surfaces) and treats humans as dead-end hosts, it provides a scalable foundation for future extensions. Incorporating environmental persistence, indirect transmission via contaminated substrates or shared resources, and explicit feedbacks between stress and immune system processes will allow application to specific disease systems such as avian influenza, Nipah virus and other bat- or bird-borne zoonoses, where stress-related ecological processes—such as resource limitation, altered host movement and changes in contact structure—are well documented [27,61]. Calibration to system-specific data using the sensitivity metrics reported here can support risk forecasting, scenario testing and intervention optimization. Moreover, the generalized framework is also readily extensible to incorporate multiple wildlife species with differing reservoir competence, habitat preferences and movement patterns. Such extensions would enable investigation of how changes in wildlife community composition influence spillover risk, including dilution effects, across heterogeneous landscapes.

Moreover, the modelling framework presented here is readily extensible to evaluate mitigation strategies, such as targeted interventions in domestic or peri-domestic animal populations, habitat management actions or behavioural modifications aimed at reducing high-risk contact. Incorporating such strategies would allow the use of multi-objective optimization approaches to explicitly explore trade-offs between competing public health, ecological and economic objectives, rather than optimizing a single outcome in isolation. For example, analyses involving the treatment of domestic animals to reduce pathogen prevalence or spillover risk would need to balance reductions in transmission against broader costs, including economic feasibility and the potential contribution to antimicrobial resistance. Embedding these considerations within a formal cost–benefit or cost–effectiveness framework would enable systematic comparison of intervention portfolios under uncertainty, supporting more transparent and context-specific decision-making in wildlife–livestock–human disease systems.

In summary, this study demonstrates that chronic stress in wildlife populations can substantially reshape zoonotic transmission dynamics. By linking ecological stressors, physiological responses and multi-host disease dynamics within a unified mathematical framework, our model bridges conceptual and quantitative approaches to spillover ecology. This integrated perspective clarifies how environmental change propagates through host condition and transmission pathways, yielding more incisive insight into the structure of spillover systems. These insights indicate that effective prevention will require coordinated, One Health–oriented strategies that integrate habitat and wildlife management with veterinary and public health interventions. Future research combining mechanistic modelling with longitudinal field data on stress physiology, contact networks and animal health has the potential to transform spillover prevention from a largely reactive effort into a predictive, quantitatively guided component of global health preparedness.

## Supplementary Material



## Data Availability

All code required to run the model and reproduce the analyses presented in this study are stored in GitHub: http://github.com/erinestafford/Wildlife_Stress_and_Spillover and have been archived within the Zenodo repository [62]. Supplementary material is available online [63].
