# Supplementary material for: Drivers of zoonotic spillover at human–domestic animal–wildlife interfaces: a multi-species model incorporating wildlife chronic stress
Source: R Soc Open Sci. 2026 Nov 4;13(11):260184. doi: 10.1098/rsos.260184 (PMC13730646; doi:10.1098/rsos.260184)
Supplement: Supplementary file 1 [file rsos.260184.zip › rsos.260184/rsos260184_si_001.pdf]

Supplementary Information for Manuscript: Drivers of Zoonotic Spillover at Human–Domestic Animal–Wildlife Interfaces: A Multi-Species Model Incorporating Wildlife Chronic Stress

Supplementary information

S1 Background literature search

Table 1. Search strings used for the systematic literature search in the Web of Science database.

Search	Category	Search terms	Records
#1	Model	control strategies OR mathematical model OR numerical simulation OR optimal control OR reproduction number OR sensitivity analysis OR transmission dynamics OR basic reproduction OR control measure OR differential equation OR model parameters OR ordinary differential OR process-based model	1,054,686
#2	Disease spillover	virus OR disease transmission OR infectious disease OR public health OR reservoir host OR human-animal interface OR zoonotic disease OR zoono* OR human infection OR human population OR zoonotic pathogen	1,739,171
#3	Stress	wildlife stress OR physiolog* OR food stress OR environment stress OR climate stress OR susceptibility OR immune response	2,094,821
#4	Exclusion	influenza OR Hepatitis OR tumor OR cancer OR Vaccination	–
#5	Year	2000 to 2024	–
#1 AND #2 AND #3 NOT #4 AND #5			1,547

S2 Additional sensitivity analyses results

S2.1 Local sensitivity indices

Table 2. Local sensitivity indices of each varied parameter for both quantities of interest.

Parameter Varied	SI: Spillover incidence (1 year)	SI: Time to first spillover
Domestic recovery rate (γ)	-0.536	0.002
Wildlife recovery rate (γ)	-0.0804	0.087

**Parameter Varied**	**SI: Spillover incidence (1 year)**	**SI: Time to first spillover**
Transmission rate: domestic → human (β)	0.234	-0.00755
Transmission rate: wildlife → human (β)	0.757	-0.218
Transmission rate: domestic → domestic (β)	0.353	-0.00139
Transmission rate: wildlife → domestic (β)	0.201	-0.00754
Transmission rate: domestic → wildlife (β)	9.302×10^{-6}	-4.500×10^{-6}
Transmission rate: wildlife → wildlife (β)	0.0465	-0.319
Initial human population (urban)	-0.00546	-0.199
Initial human population (rural)	-6.044×10^{-4}	-0.0232
Initial human population (natural)	-6.110×10^{-6}	-2.303×10^{-4}
Initial domestic population (urban)	0.177	-0.00269
Initial domestic population (rural)	0.116	-0.00178
Initial domestic population (natural)	3.368×10^{-4}	-3.298×10^{-5}
Total wildlife population	0.173	-0.128
Human–domestic contact rate (urban)	0.142	-0.00454
Human–wildlife contact rate (urban)	0.394	-0.108
Human–domestic contact rate (rural)	0.0925	-0.00301
Human–wildlife contact rate (rural)	0.256	-0.0715
Human–domestic contact rate (natural)	0	0
Human–wildlife contact rate (natural)	0.106	-0.0384
Domestic–domestic contact rate (urban)	0.213	-8.383×10^{-4}
Domestic–wildlife contact rate (urban)	0.121	-0.00454
Domestic–domestic contact rate (rural)	0.14	-5.555×10^{-4}
Domestic–wildlife contact rate (rural)	0.0794	-0.00301
Domestic–domestic contact rate (natural)	0	0
Domestic–wildlife contact rate (natural)	0	0
Wildlife–wildlife contact rate (urban)	0.0234	-0.105
Wildlife–wildlife contact rate (rural)	0.0281	-0.105
Wildlife–wildlife contact rate (natural)	0.0309	-0.616
Wildlife movement rate: urban → rural	0.0254	-0.0429
Wildlife movement rate: urban → natural	0.128	0.0786
Wildlife movement rate: rural → urban	-0.0271	0.0493
Wildlife movement rate: rural → natural	0.0997	0.132
Wildlife movement rate: natural → urban	-0.125	-0.0716
Wildlife movement rate: natural → rural	-0.0953	-0.101
Human movement rate: urban → rural	-0.00316	0.00257
Human movement rate: urban → natural	-0.00892	0.00799
Human movement rate: rural → urban	0.00476	-0.0126
Human movement rate: rural → natural	-0.00735	0.00822
Human movement rate: natural → urban	0.0013	-0.00167
Human movement rate: natural → rural	0.0147	-0.0158
Domestic movement rate: urban → rural	0.126	0.00275
Domestic movement rate: urban → natural	0	0

Parameter Varied	SI: Spillover incidence (1 year)	SI: Time to first spillover
Domestic movement rate: rural → urban	0.0387	0.00417
Domestic movement rate: rural → natural	0	0
Domestic movement rate: natural → urban	-0.07	0.0041
Domestic movement rate: natural → rural	-0.0368	0.00333
Wildlife chronic stress rate (urban)	0.00132	-0.0224
Wildlife chronic stress rate (rural)	6.199×10^{-6}	-0.00323
Wildlife chronic stress rate (natural)	-0.00825	-0.123
Wildlife stress recovery rate (urban)	-4.918×10^{-4}	0.01
Wildlife stress recovery rate (rural)	-5.724×10^{-4}	0.0855
Wildlife stress recovery rate (natural)	0.00122	0.0308
Stress scaling on transmission from wildlife	0.712	-0.263
Stress scaling on transmission to wildlife	0.00345	-0.144
Stress scaling on wildlife recovery rate	-0.0107	0.00519

S2.2 Global sensitivity indices

Table 3. Sobol sensitivity indices for SI: Incidence of spillover events in one year.

Parameter Varied	S_1 (95% CI)	S_T (95% CI)
Domestic recovery rate (γ)	0.00468 (-1.349×10^{-4}, 0.00949)	0.00802 (0.0066, 0.00944)
Wildlife recovery rate (γ)	0.0603 (0.0479, 0.0727)	0.0971 (0.0884, 0.106)
Transmission rate: domestic → human (β)	-1.801×10^{-4} (-0.00244, 0.00208)	0.00293 (0.0024, 0.00347)
Transmission rate: wildlife → human (β)	0.299 (0.267, 0.33)	0.407 (0.384, 0.43)
Transmission rate: domestic → domestic (β)	-2.453×10^{-5} (-0.00257, 0.00252)	0.00275 (0.00199, 0.00351)
Transmission rate: wildlife → domestic (β)	-8.423×10^{-5} (-7.208×10^{-4}, 5.523×10^{-4})	2.468×10^{-4} (2.032×10^{-4}, 2.904×10^{-4})
Transmission rate: domestic → wildlife (β)	6.851×10^{-6} (-1.416×10^{-6}, 1.512×10^{-5})	3.023×10^{-8} (-1.178×10^{-9}, 6.164×10^{-8})
Transmission rate: wildlife → wildlife (β)	0.0631 (0.0484, 0.0778)	0.114 (0.104, 0.125)
Initial human population (urban)	1.918×10^{-6} (-3.407×10^{-4}, 3.446×10^{-4})	5.713×10^{-5} (5.020×10^{-5}, 6.407×10^{-5})
Initial human population (rural)	4.270×10^{-6} (-5.883×10^{-5}, 6.737×10^{-5})	1.787×10^{-6} (1.447×10^{-6}, 2.127×10^{-6})
Initial human population (natural)	9.316×10^{-7} (-3.809×10^{-5}, 3.995×10^{-5})	7.459×10^{-7} (6.536×10^{-7}, 8.383×10^{-7})
Initial domestic population (urban)	-1.128×10^{-4} (-8.432×10^{-4}, 6.176×10^{-4})	2.704×10^{-4} (1.819×10^{-4}, 3.589×10^{-4})

**Parameter Varied**	S_1 **(95% CI)**	S_T **(95% CI)**
Initial domestic population (rural)	4.257×10^{-4} (-5.262×10^{-4}, 0.00138)	4.970×10^{-4} (3.576×10^{-4}, 6.364×10^{-4})
Initial domestic population (natural)	-5.786×10^{-5} (-2.184×10^{-4}, 1.026×10^{-4})	1.165×10^{-5} (8.482×10^{-6}, 1.482×10^{-5})
Total wildlife population	0.065 (0.0533, 0.0767)	0.0942 (0.0876, 0.101)
Human–domestic contact rate (urban)	1.224×10^{-5} (-0.00101, 0.00104)	5.561×10^{-4} (4.017×10^{-4}, 7.106×10^{-4})
Human–wildlife contact rate (urban)	0.0061 (0.00132, 0.0109)	0.0108 (0.00969, 0.0119)
Human–domestic contact rate (rural)	4.790×10^{-4} (-3.861×10^{-4}, 0.00134)	4.597×10^{-4} (3.199×10^{-4}, 5.995×10^{-4})
Human–wildlife contact rate (rural)	0.00356 (-0.00145, 0.00858)	0.0108 (0.0097, 0.0119)
Human–domestic contact rate (natural)	6.010×10^{-5} (-0.00115, 0.00128)	7.703×10^{-4} (5.087×10^{-4}, 0.00103)
Human–wildlife contact rate (natural)	0.0081 (0.00283, 0.0134)	0.0113 (0.00996, 0.0126)
Domestic–domestic contact rate (urban)	1.227×10^{-4} (-0.00143, 0.00168)	0.00103 (6.327×10^{-4}, 0.00142)
Domestic–wildlife contact rate (urban)	-6.274×10^{-6} (-2.269×10^{-4}, 2.144×10^{-4})	3.218×10^{-5} (2.612×10^{-5}, 3.824×10^{-5})
Domestic–domestic contact rate (rural)	3.736×10^{-4} (-9.353×10^{-4}, 0.00168)	7.729×10^{-4} (3.453×10^{-4}, 0.0012)
Domestic–wildlife contact rate (rural)	9.741×10^{-5} (-2.337×10^{-4}, 4.285×10^{-4})	3.600×10^{-5} (2.844×10^{-5}, 4.356×10^{-5})
Domestic–domestic contact rate (natural)	3.979×10^{-4} (-7.707×10^{-4}, 0.00157)	7.090×10^{-4} (3.672×10^{-4}, 0.00105)
Domestic–wildlife contact rate (natural)	-9.066×10^{-5} (-3.490×10^{-4}, 1.676×10^{-4})	3.695×10^{-5} (2.881×10^{-5}, 4.510×10^{-5})
Wildlife–wildlife contact rate (urban)	0.0147 (0.00811, 0.0213)	0.0356 (0.0309, 0.0404)
Wildlife–wildlife contact rate (rural)	0.015 (0.00562, 0.0245)	0.0407 (0.0349, 0.0464)
Wildlife–wildlife contact rate (natural)	0.0149 (0.00636, 0.0234)	0.0384 (0.0335, 0.0432)
Wildlife movement rate: urban $\rightarrow$ rural	7.129×10^{-6} (-0.00381, 0.00382)	0.0102 (0.00798, 0.0125)
Wildlife movement rate: urban $\rightarrow$ natural	0.00173 (-0.002, 0.00547)	0.01 (0.00841, 0.0116)
Wildlife movement rate: rural $\rightarrow$ urban	9.395×10^{-4} (-0.00353, 0.00541)	0.0116 (0.00963, 0.0136)
Wildlife movement rate: rural $\rightarrow$ natural	5.840×10^{-4} (-0.00327, 0.00444)	0.0111 (0.00842, 0.0137)
Wildlife movement rate: natural $\rightarrow$ urban	0.0021 (-0.00262, 0.00681)	0.0116 (0.00935, 0.0138)
Wildlife movement rate: natural $\rightarrow$ rural	0.00186 (-0.00325, 0.00698)	0.00972 (0.00797, 0.0115)

**Parameter Varied**	S_1 **(95% CI)**	S_T **(95% CI)**
Human movement rate: urban → rural	-4.247×10^{-4} (-0.00316, 0.00231)	0.00424 (0.00361, 0.00488)
Human movement rate: urban → natural	0.00298 (5.327×10^{-5}, 0.00591)	0.00467 (0.00372, 0.00562)
Human movement rate: rural → urban	0.00139 (-0.00105, 0.00383)	0.00376 (0.00318, 0.00434)
Human movement rate: rural → natural	3.670×10^{-4} (-0.00263, 0.00336)	0.00439 (0.00372, 0.00505)
Human movement rate: natural → urban	-0.00179 (-0.00471, 0.00114)	0.00449 (0.00363, 0.00534)
Human movement rate: natural → rural	-7.387×10^{-4} (-0.00413, 0.00265)	0.00499 (0.00364, 0.00634)
Domestic movement rate: urban → rural	-3.680×10^{-4} (-0.00108, 3.484×10^{-4})	3.212×10^{-4} (1.417×10^{-4}, 5.006×10^{-4})
Domestic movement rate: urban → natural	-3.274×10^{-4} (-9.418×10^{-4}, 2.869×10^{-4})	2.564×10^{-4} (1.361×10^{-4}, 3.768×10^{-4})
Domestic movement rate: rural → urban	3.736×10^{-4} (-4.374×10^{-4}, 0.00118)	2.006×10^{-4} (1.385×10^{-4}, 2.626×10^{-4})
Domestic movement rate: rural → natural	2.367×10^{-4} (-5.279×10^{-4}, 0.001)	1.726×10^{-4} (1.065×10^{-4}, 2.387×10^{-4})
Domestic movement rate: natural → urban	-7.998×10^{-5} (-5.963×10^{-4}, 4.363×10^{-4})	1.367×10^{-4} (1.006×10^{-4}, 1.727×10^{-4})
Domestic movement rate: natural → rural	-2.439×10^{-4} (-7.662×10^{-4}, 2.784×10^{-4})	2.103×10^{-4} (8.885×10^{-5}, 3.317×10^{-4})
Wildlife chronic stress rate (urban)	-2.438×10^{-4} (-8.274×10^{-4}, 3.398×10^{-4})	2.747×10^{-4} (1.540×10^{-4}, 3.954×10^{-4})
Wildlife chronic stress rate (rural)	5.069×10^{-5} (-5.826×10^{-4}, 6.839×10^{-4})	2.331×10^{-4} (1.543×10^{-4}, 3.120×10^{-4})
Wildlife chronic stress rate (natural)	-2.928×10^{-4} (-9.235×10^{-4}, 3.379×10^{-4})	2.788×10^{-4} (1.543×10^{-4}, 4.033×10^{-4})
Wildlife stress recovery rate (urban)	-2.857×10^{-5} (-6.280×10^{-4}, 5.709×10^{-4})	2.667×10^{-4} (8.703×10^{-5}, 4.464×10^{-4})
Wildlife stress recovery rate (rural)	-3.796×10^{-4} (-7.808×10^{-4}, 2.151×10^{-5})	1.310×10^{-4} (1.074×10^{-4}, 1.547×10^{-4})
Wildlife stress recovery rate (natural)	-2.590×10^{-4} (-8.118×10^{-4}, 2.937×10^{-4})	1.476×10^{-4} (1.225×10^{-4}, 1.727×10^{-4})
Stress scaling on transmission from wildlife	0.175 (0.155, 0.194)	0.246 (0.231, 0.262)
Stress scaling on transmission to wildlife	0.00723 (5.948×10^{-4}, 0.0139)	0.0245 (0.0211, 0.028)
Stress scaling on wildlife recovery rate	0.0431 (0.0314, 0.0548)	0.0813 (0.0735, 0.089)

Table 4. Sobol sensitivity indices for SI: Time to first spillover event.

**Parameter Varied**	S_1 **(95% CI)**	S_T **(95% CI)**
Domestic recovery rate (γ)	-0.00136 (-0.0105, 0.0078)	0.0241 (0.0107, 0.0376)
Wildlife recovery rate (γ)	0.0585 (0.0296, 0.0874)	0.421 (0.372, 0.47)
Transmission rate: domestic → human (β)	5.928×10^{-5} (-8.853×10^{-6}, 1.274×10^{-4})	1.309×10^{-5} (-2.814×10^{-6}, 2.900×10^{-5})
Transmission rate: wildlife → human (β)	-6.244×10^{-4} (-0.00149, 2.399×10^{-4})	0.00421 (-7.095×10^{-4}, 0.00913)
Transmission rate: domestic → domestic (β)	0.00236 (-0.0049, 0.00962)	0.00885 (0.00154, 0.0162)
Transmission rate: wildlife → domestic (β)	-2.055×10^{-5} (-9.239×10^{-5}, 5.129×10^{-5})	1.886×10^{-6} (1.667×10^{-7}, 3.605×10^{-6})
Transmission rate: domestic → wildlife (β)	1.874×10^{-5} (-1.986×10^{-5}, 5.735×10^{-5})	8.684×10^{-6} (-8.864×10^{-6}, 2.623×10^{-5})
Transmission rate: wildlife → wildlife (β)	0.139 (0.0981, 0.18)	0.595 (0.537, 0.654)
Initial human population (urban)	-1.618×10^{-4} (-6.611×10^{-4}, 3.375×10^{-4})	0.00177 (-0.00174, 0.00529)
Initial human population (rural)	-1.877×10^{-4} (-5.982×10^{-4}, 2.229×10^{-4})	9.728×10^{-4} (-0.00102, 0.00296)
Initial human population (natural)	1.805×10^{-5} (-1.302×10^{-5}, 4.912×10^{-5})	5.861×10^{-6} (-4.645×10^{-6}, 1.637×10^{-5})
Initial domestic population (urban)	3.245×10^{-5} (-0.00104, 0.00111)	0.00425 (-3.540×10^{-4}, 0.00885)
Initial domestic population (rural)	-5.484×10^{-4} (-0.0016, 5.057×10^{-4})	0.00492 (1.959×10^{-4}, 0.00965)
Initial domestic population (natural)	6.951×10^{-4} (-9.414×10^{-4}, 0.00233)	8.168×10^{-5} (-9.925×10^{-5}, 2.626×10^{-4})
Total wildlife population	0.00468 (-0.00964, 0.019)	0.165 (0.134, 0.196)
Human–domestic contact rate (urban)	3.509×10^{-5} (-3.726×10^{-5}, 1.074×10^{-4})	3.826×10^{-7} (-3.119×10^{-8}, 7.964×10^{-7})
Human–wildlife contact rate (urban)	2.398×10^{-5} (-2.250×10^{-5}, 7.045×10^{-5})	1.288×10^{-5} (-2.007×10^{-6}, 2.776×10^{-5})
Human–domestic contact rate (rural)	-1.930×10^{-5} (-6.572×10^{-5}, 2.712×10^{-5})	7.065×10^{-7} (9.484×10^{-8}, 1.318×10^{-6})
Human–wildlife contact rate (rural)	4.252×10^{-5} (-3.059×10^{-5}, 1.156×10^{-4})	3.419×10^{-5} (-1.573×10^{-5}, 8.411×10^{-5})
Human–domestic contact rate (natural)	-2.091×10^{-5} (-6.222×10^{-5}, 2.041×10^{-5})	6.059×10^{-6} (-1.665×10^{-6}, 1.378×10^{-5})
Human–wildlife contact rate (natural)	4.475×10^{-5} (-3.721×10^{-5}, 1.267×10^{-4})	3.551×10^{-5} (-3.305×10^{-5}, 1.041×10^{-4})
Domestic–domestic contact rate (urban)	0.00155 (-0.00561, 0.00871)	0.0111 (0.00173, 0.0205)
Domestic–wildlife contact rate (urban)	-2.032×10^{-4} (-6.134×10^{-4}, 2.070×10^{-4})	9.708×10^{-4} (-0.00102, 0.00296)

**Parameter Varied**	S_1 **(95% CI)**	S_T **(95% CI)**
Domestic–domestic contact rate (rural)	0.003 (-0.0042, 0.0102)	0.0069 (2.980×10^{-4}, 0.0135)
Domestic–wildlife contact rate (rural)	-9.272×10^{-6} (-2.553×10^{-5}, 6.985×10^{-6})	1.280×10^{-6} (-3.122×10^{-7}, 2.873×10^{-6})
Domestic–domestic contact rate (natural)	0.00217 (-0.00905, 0.0134)	0.0138 (0.00446, 0.0231)
Domestic–wildlife contact rate (natural)	1.331×10^{-5} (-1.620×10^{-5}, 4.281×10^{-5})	4.713×10^{-6} (-4.503×10^{-6}, 1.393×10^{-5})
Wildlife–wildlife contact rate (urban)	0.00572 (-0.0141, 0.0255)	0.213 (0.175, 0.25)
Wildlife–wildlife contact rate (rural)	0.0132 (-0.0146, 0.041)	0.264 (0.223, 0.304)
Wildlife–wildlife contact rate (natural)	0.0197 (-0.0116, 0.0511)	0.245 (0.205, 0.285)
Wildlife movement rate: urban → rural	-0.00618 (-0.0159, 0.00349)	0.0819 (0.0598, 0.104)
Wildlife movement rate: urban → natural	-0.00233 (-0.00925, 0.00459)	0.0656 (0.0444, 0.0869)
Wildlife movement rate: rural → urban	-8.024×10^{-4} (-0.0179, 0.0163)	0.0814 (0.0607, 0.102)
Wildlife movement rate: rural → natural	-0.00645 (-0.0205, 0.00764)	0.089 (0.0679, 0.11)
Wildlife movement rate: natural → urban	1.979×10^{-4} (-0.00966, 0.0101)	0.0954 (0.0689, 0.122)
Wildlife movement rate: natural → rural	-0.00499 (-0.0198, 0.00977)	0.0986 (0.075, 0.122)
Human movement rate: urban → rural	-1.889×10^{-4} (-5.764×10^{-4}, 1.987×10^{-4})	8.969×10^{-4} (-9.025×10^{-4}, 0.0027)
Human movement rate: urban → natural	-4.280×10^{-6} (-2.245×10^{-5}, 1.389×10^{-5})	1.671×10^{-6} (-1.376×10^{-7}, 3.479×10^{-6})
Human movement rate: rural → urban	-2.069×10^{-4} (-6.195×10^{-4}, 2.057×10^{-4})	9.733×10^{-4} (-0.00102, 0.00296)
Human movement rate: rural → natural	-9.462×10^{-6} (-2.343×10^{-5}, 4.510×10^{-6})	4.806×10^{-7} (1.583×10^{-7}, 8.029×10^{-7})
Human movement rate: natural → urban	2.594×10^{-5} (-7.502×10^{-6}, 5.937×10^{-5})	5.502×10^{-6} (-3.429×10^{-6}, 1.443×10^{-5})
Human movement rate: natural → rural	-9.186×10^{-6} (-4.544×10^{-5}, 2.707×10^{-5})	7.360×10^{-6} (-4.104×10^{-6}, 1.883×10^{-5})
Domestic movement rate: urban → rural	2.570×10^{-5} (-6.069×10^{-4}, 6.583×10^{-4})	0.00293 (-9.035×10^{-4}, 0.00675)
Domestic movement rate: urban → natural	2.968×10^{-4} (-4.744×10^{-4}, 0.00107)	0.00444 (-0.00105, 0.00993)
Domestic movement rate: rural → urban	4.210×10^{-5} (-9.818×10^{-4}, 0.00107)	0.00672 (-3.718×10^{-4}, 0.0138)
Domestic movement rate: rural → natural	-4.694×10^{-4} (-0.00119, 2.543×10^{-4})	0.00273 (-0.0016, 0.00706)

**Parameter Varied**	S_1 **(95% CI)**	S_T **(95% CI)**
Domestic movement rate: natural → urban	-4.685×10^{-4} (-0.00143, 4.912×10^{-4})	0.00603 (1.138×10^{-4}, 0.0119)
Domestic movement rate: natural → rural	-7.020×10^{-4} (-0.00171, 3.083×10^{-4})	0.00642 (4.872×10^{-4}, 0.0124)
Wildlife chronic stress rate (urban)	5.650×10^{-4} (-3.934×10^{-4}, 0.00152)	0.00626 (7.996×10^{-4}, 0.0117)
Wildlife chronic stress rate (rural)	-0.00111 (-0.00261, 3.880×10^{-4})	0.0141 (0.0062, 0.0219)
Wildlife chronic stress rate (natural)	4.173×10^{-4} (-7.963×10^{-4}, 0.00163)	0.0108 (0.00376, 0.0178)
Wildlife stress recovery rate (urban)	-1.514×10^{-4} (-0.00154, 0.00124)	0.0144 (0.00529, 0.0235)
Wildlife stress recovery rate (rural)	-0.00137 (-0.00275, 1.107×10^{-6})	0.0115 (0.0038, 0.0192)
Wildlife stress recovery rate (natural)	-8.412×10^{-4} (-0.00208, 4.017×10^{-4})	0.0132 (0.00402, 0.0225)
Stress scaling on transmission from wildlife	0.0238 (0.00731, 0.0404)	0.288 (0.244, 0.332)
Stress scaling on transmission to wildlife	0.0207 (-0.00174, 0.0431)	0.305 (0.262, 0.348)
Stress scaling on wildlife recovery rate	0.0329 (0.00125, 0.0646)	0.473 (0.424, 0.522)
